# Continuous Local Antibiotics Perfusion Therapy for Acute Deep Infections after Open Fractures

**DOI:** 10.1155/2022/2563939

**Published:** 2022-01-18

**Authors:** Shunsuke Takahara, Akihiro Maruo, Hiroyuki Takayama, Toshihiko Harada

**Affiliations:** ^1^Department of Orthopaedic Surgery, Hyogo Prefectural Kakogawa Medical Center, Kakogawa, Japan; ^2^Department of Orthopaedic Surgery, Steel Memorial Hirohata Hospital, Himeji, Japan

## Abstract

Unresolved bone and soft tissue infections remain a great hindrance to fracture management worldwide, both economically and functionally for the patient. For this purpose, the benefits of local antibiotic administration besides systemic therapy have been elucidated. We present a retrospective descriptive analysis of six patients (4 males and 2 females) with acute deep infections after open fractures managed using the continuous local antibiotic perfusion (CLAP) therapy. After sufficient debridement, gentamicin solution concentrated at 1,200 *μ*g/mL was continuously infused (2 mL/h) for 7–12 days by syringe pump through an inlet tube placed on the infected area. The antibiotics injected into the infected area were both collected and perfused by negative pressure using a negative-pressure wound therapy system. After an average of 9.5 days of CLAP therapy, symptoms of infection disappeared, and the bacterial culture was negative. There were no cases of recurrence during the follow-up period, and no complications, such as acute renal failure, ototoxicity, allergic or hypersensitivity reactions, and impaired fracture healing, were observed. All six cases were successfully managed with the CLAP therapy without any serious side effects. CLAP therapy may be a potential treatment option for acute deep infections after open fractures.

## 1. Introduction

Bone and soft tissue infections after an injury continue to remain a worldwide concern, especially in the case of open fractures, which are known to be associated with a high risk for infection even when treated appropriately. Once infection occurs, the subsequent treatment is often impeded due to drug intolerance that results from the formation of a biofilm [1]. The overall success rate of treatment against orthopedic infections varies between 57% and 88% [2], incurring significant medical costs and the risk of long-time disability for the patient [3]. Therefore, it is desirable to establish a therapy suitable for clinical settings that is less invasive and economic while offering a higher success rate.

Successful eradication of potential fracture-related infections requires dissecting the affected tissue, removing loose implants and foreign bodies, creating a stable environment for the fracture, dead space management, and antibiotic therapy [4]. Usually, systemic antibiotics are administered against fracture-related infections in trauma surgeries. Often, the target tissue of such systemically administered antibiotics is a surgical or an open wound that itself involves tissue disruption and impaired blood supply. Therefore, systemic antibiotic therapy may lack effective distribution to the infected area after open fractures.

Over the last few decades, the benefits of local antibiotic administration along with systemic therapy have been reported in the prevention and management of bone and soft tissue infections as the antibiotics are placed directly within the infected area [5]. Moreover, local administration allows for higher local concentrations with lesser total drug volumes, which improves the overall effect of the antimicrobial agent while reducing the risk of systemic toxicity [5]. In a review, Winkler et al. described that one of the prerequisites for the eradication of biofilm-mediated infections was the elimination of sessile bacteria existing inside the fracture fragments using antimicrobial substances in consistently high concentrations [6]. For this reason, we employed a novel local antibiotic therapy at our institution to manage acute deep infections after open fractures. We report a series of cases to demonstrate the efficacy and safety of our novel continuous local antibiotic perfusion (CLAP) therapy [7, 8] applied to acute deep infections after open fractures.

## 2. Case Series

### 2.1. Continuous Local Antibiotics Perfusion (CLAP) Therapy

**Figure 1:**
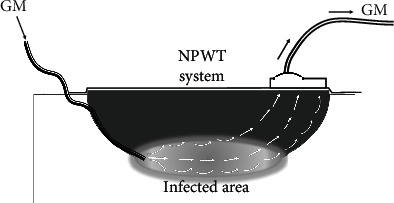
The schema of continuous local antibiotic perfusion (CLAP) therapy. A high concentration of gentamicin solution injected from the inlet tube into the infected area is perfused by the negative pressure wound therapy (NPWT) system. GM: gentamicin.

After obtaining ethical approval from the Institutional Review Board (registration number -201908), we applied the CLAP therapy for acute deep infection after open fractures. As against the local placement of antibiotic-containing substances, CLAP therapy involves only topical infusion of an antibiotic solution. After sufficient debridement, a 6 fr inlet tube (Atom Tube, Atom Medical, Tokyo, Japan) was placed on the infected area to continuously inject the antibiotic solution. A negative-pressure wound therapy (NPWT) system (Renasys; Smith & Nephew Medical Ltd., Kingston Upon Hull, UK) was set aside from the inlet tube to collect the antibiotics injected into the infected area and perfuse them by negative pressure (Figure 1).

Sixty milligrams of gentamicin diluted in 50 mL of normal saline (1,200 *μ*g/mL) was continuously infused (2 mL/h) immediately after the surgery by a syringe pump through the inlet tube. In case of a large infected area, one to three inlet tubes were placed and a continuous infusion of gentamicin solution was ensured for each. The administered antibiotic solution was perfused by suction tube or NPWT system at a negative pressure of 40 mmHg to ensure that fresh; high-concentration antibiotics are delivered to the target areas. Wound dressing, including the NPWT system, was changed once or twice a week; postoperative rehabilitation was initiated without any restrictions associated with CLAP therapy. Both systemic and local antibiotic administrations are continued for approximately 1–2 weeks and terminated when infectious findings, such as C-reactive protein, localized fever, and swelling and showed an obvious reduction. We removed the NPWT system when delayed closure or wound bed preparation for the skin graft was completely achieved.

We retrospectively evaluated six patients (four males, two females) who received the CLAP therapy from their case notes.  Table 1 shows the characteristics of the current case series. The average age of the patients was 45.7 years (range: 19–91 years). All cases were diagnosed as acute deep infections following Gustilo type III open fractures. Deep infections were assigned according to the 1999 Centers for Disease Control and Prevention guidelines [9]; wound bacterial cultures were positive in all cases. The average time of occurrence of the infection after the initial injury was 11.8 days (range: 2–29 days), and the average follow-up period was 36 months (range: 18–46).

All patients received the CLAP therapy combined with sufficient debridement and systemic antibiotics (based on wound culture) as soon as a deep infection was confirmed. The infection symptoms (reddish, local heat, swelling, and discharge) disappeared with negative bacterial cultures after an average of 9.5 days of CLAP therapy, and there were no cases of recurrence during the follow-up period (Table 2). A summary of renal function and maximum serum levels of gentamicin is shown in Table 3. No complications, such as acute renal failure, ototoxicity, allergic or hypersensitivity reactions, or impaired fracture healing, were observed.

### 2.2. Representative Case Presentation

#### 2.2.1. Case 1 (Table 1): Open Calcaneal Fracture

**Figure 2:**
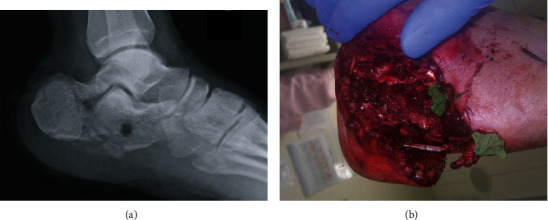
(a) Lateral view of the initial radiograph revealed a tongue-type calcaneus fracture. (b) Severe contamination by standing water of a 10 cm wound on the lateral side of the calcaneus; small leaves were attached to the wound.

**Figure 3:**
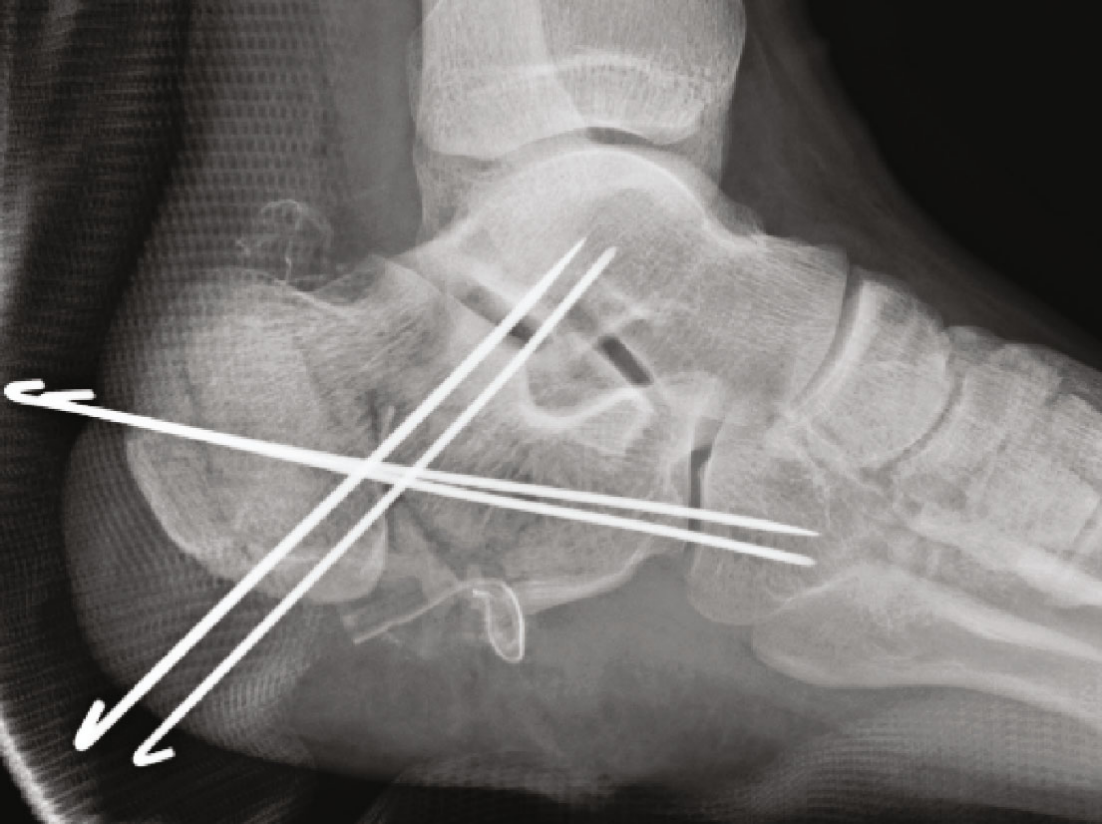
Irrigation, debridement, and pinning by four Kirschner wires were performed following the trauma resuscitation. The wound was closed.

**Figure 4:**
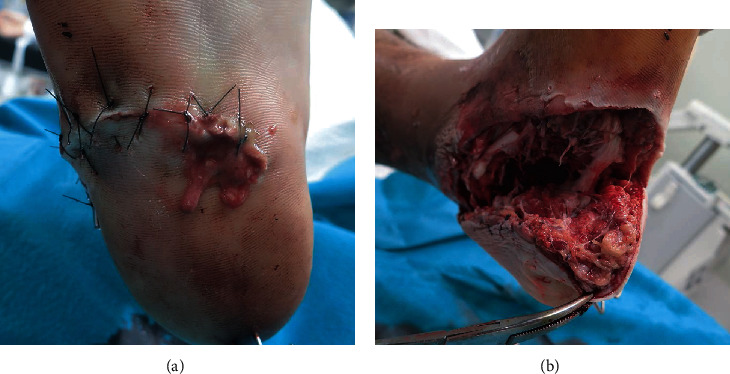
Two days after the injury. (a) Obvious signs of wound infection including pus, spreading redness, and increased swelling. (b) Irrigation and debridement were performed, and the wound was left open.

A 22-year-old male patient presented at our emergency department after a motorcycle accident. Multiple injuries, including an open right calcaneal fracture, pelvic ring fracture, right renal artery injury, and inferior vena cava injury, with vital signs corresponding to shock, were detected. The right calcaneal open fracture with a 10 cm wound on the lateral side of the calcaneus was markedly contaminated by standing water (Gustilo IIIA, Figure 2). Prompt irrigation, debridement, and pinning were performed for the calcaneal fracture after successful trauma resuscitation (Figure 3). Two days after the injury, the cause of the infected wound was noted to be *Aeromonas hydrophila*. Irrigation and debridement were repeated subsequently, and the wound was kept opened (Figure 4).

**Figure 5:**
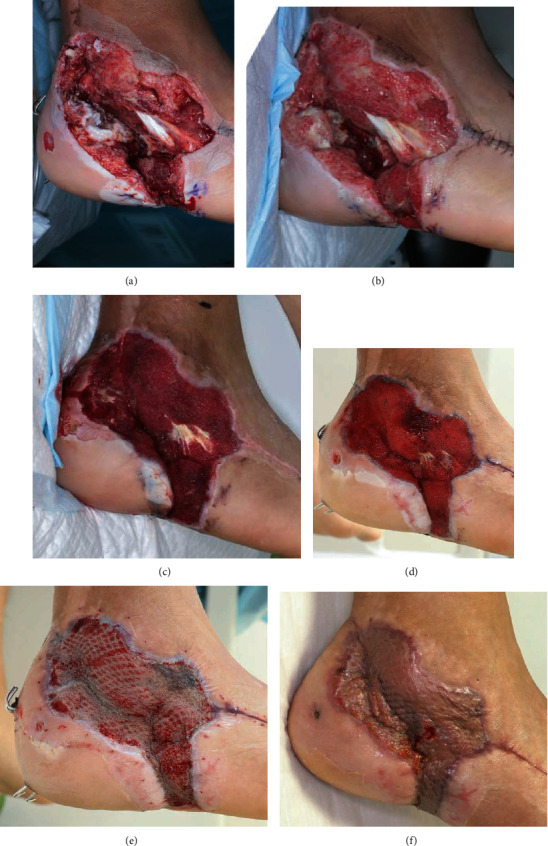
(a) At postinjury day (PID) 6, pus was still pooled in the wound. (b) PID-14—signs of infection persisted, and continuous local antibiotic perfusion (CLAP) therapy was initiated. (c) PID-19 (5 days after initiation of CLAP therapy)—the signs of infection disappeared, and a well-vascularized granulation tissue growth was observed. (d) PID-36—wound bed preparation was almost complete. (e) PID-47 (6 days after split-thickness skin grafting). (f) PID-64—the infected wound was completely cured.

**Figure 6:**
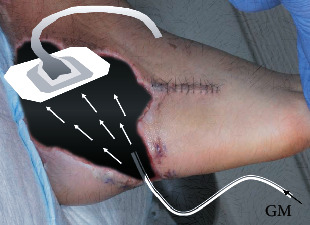
Schema of continuous local antibiotics perfusion circuits adapted to case #1. GM: gentamicin.

**Figure 7:**
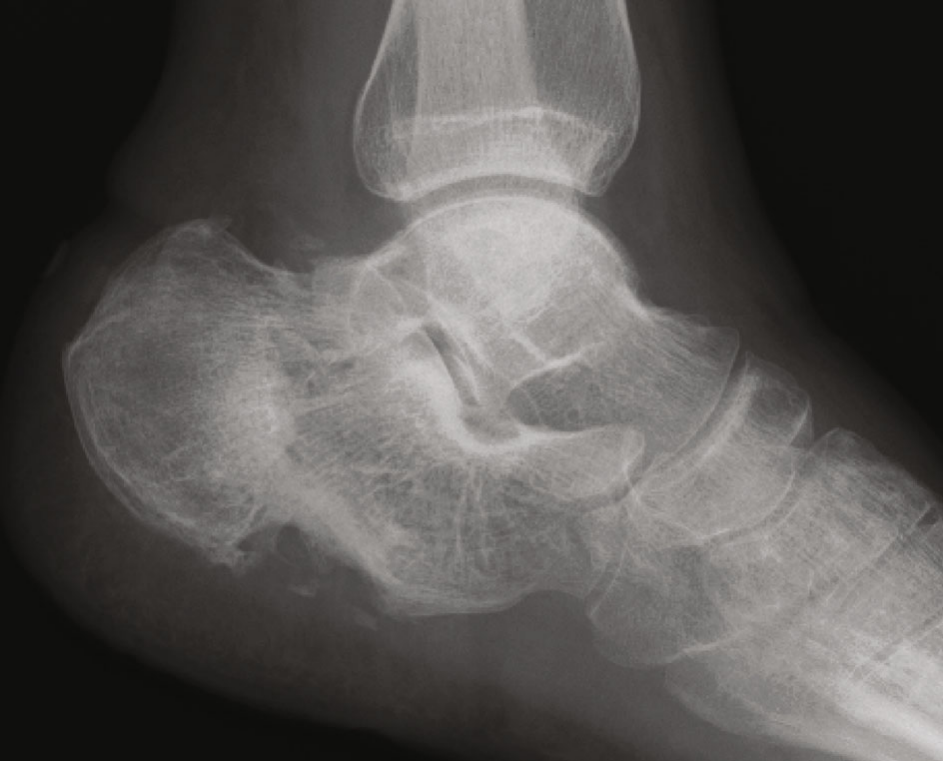
Three months after the injury, successful bone healing without any symptoms was achieved.

Despite daily irrigation and debridement at the bedside, the wound infection remained uncontrolled, and the calcaneus was exposed (Figure 5(a)). Therefore, we introduced the CLAP therapy 14 days after the injury; immediate favorable results in the form of good infection control and formation of new granulation tissue over the calcaneus and peroneal tendon were observed (Figures 5(b) and 5(c)). Subsequently, when the wound bed was sufficiently prepared, split-thickness skin grafting was performed six weeks after the injury and the inlet tube and NPWT system were placed (Figures 5(d)–5(f)). In this case, the inlet tube and NPWT system were placed as shown in Figure 6. Eight weeks after the injury, the K-wires were extracted, and a bone union was finally obtained without recurrence of infection (Figure 7). There was no evidence of renal failure or ototoxicity, and the patient was able to walk without pain at the final follow-up (three years after the injury).

## 3. Discussion

We present a case series of CLAP therapy, a novel drug delivery system that ensures consistent concentrated local antibiotic administration. CLAP therapy, originally proposed by Maruo in 2020, has recently been recognized as a treatment option for bone and soft tissue infections in Japan [7, 8]. Himeno et al. reported about the successful treatment of patients with severe infections of the lower limb, including abscesses and osteomyelitis, using CLAP [7]. Similarly, Takahashi et al. used CLAP in four patients who developed SSIs after an instrumented spinal surgery and reported that CLAP controlled the infections without any severe adverse events and enabled implant retention in all cases [10]. In our case series as well, the infections subsided quickly in all six patients treated with CLAP therapy, and no complications were observed.

Buchholz and Engelbrecht first described the use of antibiotic-loaded polymethyl methacrylate (PMMA) in 1970 [11]; since then, PMMA has traditionally been used to prevent and treat bone and soft tissue infections. Local administration of antibiotics offers the advantage of ensuring ample amounts of high drug concentration to be delivered directly to the infection site, which is not possible by systemic administration alone. Although antibiotic-loaded PMMA is commonly used for local antibiotic administration, the extent of antibiotic elution remains uncertain. Some drug elution studies have revealed that PMMA initially released antibiotics in high concentrations but the amount declined quickly to ineffective concentrations in 4–6 weeks [12]. Moreover, when the release of antibiotics falls below an effective concentration, PMMA becomes a foreign substance and needs to be removed. Furthermore, despite its action of eluting the antibiotic, PMMA may act as a biomaterial surface to which bacteria can preferentially adhere, grow, and potentially develop biofilm [13]. Over the recent years, a drug delivery system using absorbable materials as carriers has been developed [14]. Further, new drug delivery methods that do not require carriers, such as injecting an antibiotic solution directly into the wound, have been proposed [15]. CLAP is one such method that allows continuous perfusion of high concentrations of antibiotics into infected tissues, maximizing the concentration-dependent properties of gentamicin to control infection.

One of the primary causes of refractory infections in orthopedic injuries is the biofilm produced by the bacteria, hindering the action of the antibiotic agents on the bacteria encased in the biofilm, especially in orthopedic trauma surgery, where a biofilm can easily form around the implant impeding infection control [1, 16]. Bacterial susceptibility to antibiotics is usually assessed using the minimum inhibitory concentration (MIC) of a drug. In the case of biofilm formation, a minimum biofilm eradication concentration (MBEC) is required for antibiotic action, which is usually 100–1000 times higher than the MIC [17], and it is not possible to achieve the desired local MBEC antimicrobial concentrations by systemic administration alone. Among the bacteria detected in our case series, the usual MBEC for methicillin-sensitive *Staphylococcus aureus* (MSSA) to gentamicin is reported as approximately 1,000 *μ*g/mL [18]. In the CLAP therapy, we could deliver 1,200 *μ*g/mL of gentamicin, which exceeded the MBEC for the MSSA, directly to the wound, which may have contributed to the prompt infection control. Although there is no data regarding MBECs of the other bacteria to gentamicin, we assumed that the concentrations were effective against other bacteria as well, which was corroborated by the disappearance of bacterial cultures after the CLAP therapy. Nevertheless, a comprehensive search for MBECs against various bacterial species is warranted.

Another advantage of local antibiotic therapy is that it is relatively safe and less likely to cause systemic complications. In our method, locally administered antibiotics are both delivered to and collected back from the infected area using negative pressure from the NPWT system to avoid a localized pooling of high concentrations of antimicrobials. Nevertheless, a small amount of antibiotics has been noted to migrate into the bloodstream; therefore, close monitoring of the blood levels and measurement of renal function can help ensure its safety.

There are several limitations to this study. First, a descriptive analysis of a small sample of patients was done retrospectively without comparing with other local antibiotic therapies. The patient characteristics, including the site of infection and the method of osteosynthesis, were different. Therefore, to confirm the efficacy and safety of our local therapy, a large-sample prospective randomized controlled study is required. Second, as with other local antibiotic therapies such as antibiotic-loaded PMMA, the biodistribution of the administered antibiotics has not been clarified. Further in vivo studies are warranted to demonstrate the local efficacy of antibiotics.

## 4. Conclusions

Using a novel method of continuous local infusion of antibiotics, the CLAP therapy, we were able to successfully manage acute deep infections after open fractures in our patients. The current case series substantiated the role of CLAP as a local antibiotic therapy for treating deep infections involving the bone and soft tissues in orthopedic trauma.

## Figures and Tables

**Table 1:** Characteristics of current series of patients with musculoskeletal infection, treated with CLAP.

Patient number	Age/gender	Location	Causation	Osteosynthesis	Implant care	Microbiology	Time to occurrence of infection after initial surgery or injury (days)
1	22/male	Calcaneus	G3A open fracture	Pinning	Retention	*A. hydrophila*	2
2	19/male	Distal tibia	G3C open fracture	External fixation	Retention	*Corynebacterium*	21
3	35/male	Distal tibia	G3A open fracture	Plate fixation	Retention	MSSA	7
4	48/female	Foot	G3B open fracture-dislocation	Conservative	Not applicable	MSSA	7
5	59/female	Ankle	G3A open fracture-dislocation	Plate fixation	Retention	MSSA	29
6	91/male	Patellar	G3B open fracture	Conservative	Not applicable	*S. maltophilia* *A. hydrophila*	5

CLAP: continuous local antibiotic perfusion; G3: Gustilo type 3; *A. hydrophila*: *Aeromonas hydrophila*; MSSA: methicillin-sensitive *Staphylococcus aureus*; *S. maltophilia*: *Stenotrophomonas maltophilia.*

**Table 2:** Summary of management and clinical results.

Patient number	Duration of CLAP (days)	Surgical intervention	Systemic antibiotics	CRP (mg/dL) Pre^∗^	CRP (mg/dL) Post^†^	Outcomes		Follow-up (months)
						Recurrence	Bone union	
1	12	Debridement	TAZ/PIPC, 15 days	4.20	2.84	No	Yes	45
2	10	Debridement	MINO, 10 days	0.57	0.12	No	Yes	45
3	8	Debridement	CEZ, 11 days	0.64	0.13	No	Yes	46
4	7	Debridement	SBT/ABPC, 8 days	1.96	0.76	No	Yes	20
5	9	Debridement	CEZ, 29 days	8.40	0.44	No	Yes	18
6	11	Debridement	SBT/ABPC, 21 days	5.46	4.22	No	Yes	43
Average	9.5			3.54	1.42			36

CLAP: continuous local antibiotic perfusion; CRP: C-reactive protein; TAZ/PIPC: tazobactam/piperacillin; MINO: minocycline; CEZ: cefazolin; SBT/ABPC: sulbactam/ampicillin; ^∗^“Pre” indicates immediately before starting CLAP therapy. ^†^“Post” indicates within two days after finishing CLAP therapy.

**Table 3:** Summary of renal function and maximum serum level of gentamicin.

Patient number	Cre (mg/dL) Pre^∗^	Cre (mg/dL) Post^†^	BUN (mg/dL) Pre^∗^	BUN (mg/dL) Post^†^	eGFR (mL/min/1.73m^2^) Pre^∗^	eGFR (mL/min/1.73m^2^) Post^†^	Maximum serum level of gentamicin (*μ*g/mL)
1	0.67	0.60	11	11	123.8	139.7	Not tested
2	0.71	0.81	20	16	121.2	104.9	Not tested
3	0.7	0.63	12	9	103.3	115.9	0.6
4	0.76	0.80	12	12	63.7	60.2	0.4
5	0.45	0.40	12	15	106.6	121.2	0.2
6	0.63	0.59	16	15	88.1	94.7	0.9
Average	0.65	0.64	13.8	13.0	101.1	106.1	0.525

Cre: creatinine; BUN: blood urea nitrogen; eGFR: estimated glomerular filtration rate; ^∗^“Pre” indicates immediately before starting CLAP therapy. ^†^“Post” indicates within two days after finishing CLAP therapy.

## Data Availability

The clinical data used to support the findings of this study are available from the corresponding author upon request.
